# Supplementary material for: The Evolution and Characterization of the RNA Interference Pathways in Lophotrochozoa
Source: Genome Biol Evol. 2024 May 7;16(5):evae098. doi: 10.1093/gbe/evae098 (PMC11114477; doi:10.1093/gbe/evae098)
Supplement: evae098_Supplementary_Data [file evae098_supplementary_data.zip › Supplementary_materials.zip!/selected_miRNAs/NoGe_selected_miRNAs/known_miRNA_families_pdf_files/NMRB01001162.1_Notospermus_geniculatus_strain_Ushimado_scaffold1162,_whole_genome_shotgun_sequence_25710.pdf]

Provisional ID	:	NMRB01001162.1_Notospermus_geniculatus_strain_Ushimado_scaffold1162,_whole_genome_shotgun_sequence_25710
Score total	:	106804
Score for star read(s)	:	3.9
Score for read counts	:	106795.2
Score for mfe	:	1.8
Score for randfold	:	0
Score for cons. seed	:	3
Total read count	:	209486
Mature read count	:	209327
Loop read count	:	0
Star read count	:	159

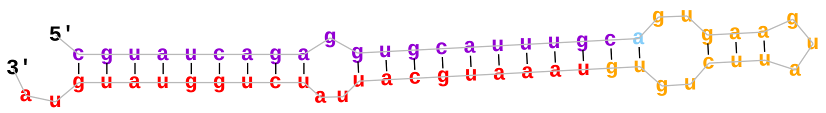

Genome coordinates: NMRB01001162.1 186352-186410

pre-miRNA sequence:
CGUAUCAGAGGUGCAUUUGCCGUAUCAGAGGUGCAUUUGCAGUGAAGUAUUCUGUGUAAAUGCAUUAUCUGGUAUGUA

Mature sequence: UAAAUGCAUUAUCUGGUAUGUA

Aligned mature sequence of the *N. geniculatus* pre-miRNA with the top 5 hits from the BLAST search against the pre-miRNA mirGeneDB set:

```
                                 *********************
    Nge_Mir-277-P1               uaaaugcauuaucugguaugua-
    Esc-Mir-277_3p               uaaaugcauuaucugguaugug-
    Npo-Mir-277_3p               uaaaugcauuaucugguaugug-
    Obi-Mir-277_3p               uaaaugcauuaucugguauguga
    Ovu-Mir-277_3p               uaaaugcauuaucugguauguga
    Cte-Mir-277-P1_3p            uaaaugcauuaucugguaugua-
```
